# Atogepant after anti-CGRP monoclonal antibodies failure in migraine: a multicenter real-world study of effectiveness, safety, persistence and predictors of response

**DOI:** 10.1186/s10194-025-02239-1

**Published:** 2025-11-28

**Authors:** Albert Muñoz-Vendrell, Sergio Campoy-Díaz, Paloma Valín-Villanueva, Javier Casas-Limón, Iris Fernández-Lázaro, Nuria González-García, Sonia Santos-Lasaosa, Yésica González Osorio, Alicia Gonzalez-Martinez, Jaume Campdelacreu, Leonardo Portocarrero-Sánchez, Luis Miguel Cano Sánchez, Sonia María García Sánchez, Alba Pérez-de-la-parte, Noemí Morollón Sánchez-Mateos, Alba López-Bravo, Ane Mínguez-Olaondo, Antonio Sánchez-Soblechero, Alberto Lozano Ros, Cristian Morales Hernández, Alberto Andrés López, Almudena Layos-Romero, Edoardo Caronna, Marta Torres-Ferrús, Alicia Alpuente, Patricia Pozo-Rosich, Robert Belvís, David Garcia-Azorin, Javier Díaz-de-Terán, Ángel Luis Guerrero-Peral, Ana Beatriz Gago-Veiga, Mariano Huerta-Villanueva

**Affiliations:** 1 Headache Unit, Neurology Department, Hospital Universitari de Bellvitge-IDIBELL, Universitat de Barcelona, L’Hospitalet de Llobregat, Barcelona, Spain; 2 Neurology Department, Hospital de Viladecans-IDIBELL, Viladecans, Barcelona, Spain; 3 Headache Unit, Neurology Department, Hospital Universitario Fundación Alcorcón, Madrid, Spain; 4 Headache Unit, Neurology Department, Instituto de Investigación Sanitaria, Hospital Universitario de la Princesa, Madrid, Spain; 5 Headache Unit, Neurology Department, Hospital Universitario San Carlos, Madrid, Spain; 6 Universidad de Zaragoza, IIS Aragón, Zaragoza, Spain; 7 Neurología, HCU Lozano Blesa de Zaragoza, Zaragoza, Spain; 8 Headache Unit, Neurology Department, Hospital Clínico Universitario, Valladolid Biosanitary Research Institute (IBIoVALL), Valladolid, Spain; 9 Servicios de Neurología e Inmunología, Hospital Universitario de la Princesa e Instituto de Investigación Sanitaria Princesa (IIS-Princesa), Madrid, Spain; 10 Headache Unit, Neurology Department, La Paz University Hospital and Institute for Health Research – IdiPAZ (La Paz University Hospital, Universidad Autónoma de Madrid), Madrid, Spain; 11 Neurology Department, Hospital Sant Joan Despí, Consorci Sanitari Integral, Barcelona, Spain; 12 Headache Unit, Department of Neurology, Hospital Universitario Río Hortega de Valladolid, Valladolid, Spain; 13 Headache and Neuralgia Unit, Hospital de la Santa Creu i Sant Pau, Barcelona, Spain; 14 Neurology Department, Hospital Reina Sofia, Tudela, Navarra, Spain; 15 Aragon Institute for Health Research (IIS Aragón), Zaragoza, Spain; 16 Department of Health Sciences, Public University of Navarra, Tudela, Spain; 17 Neurology Department, Hospital Universitario Donostia, Donostia-San Sebastián, Spain; 18 Área de Neurociencias, Instituto de Salud Biogipuzkoa, Donostia-San Sebastián, Spain; 19 Facultad de Ciencias de la Salud, Universidad de Deusto, Donostia-Bilbo, Spain; 20 Headache Unit, Neurology Department, Hospital General Universitario Gregorio Marañón, Madrid, Spain; 21 Neurology Department, Hospital Universitario de Canarias, Santa Cruz de Tenerife, Spain; 22 Headache Unit, Neurology Department, Hospital General Universitario de Albacete, Albacete, Spain; 23 Headache Clinic, Neurology Department, Vall d’Hebron Hospital, Barcelona, Spain; 24 Headache and Neurological Pain Research Group, VHIR, Department of Medicine, Universitat Autònoma de Barcelona, Barcelona, Spain; 25 Departament of Medicine, University of Valladolid (UVA), Valladolid, Spain

**Keywords:** Atogepant, Anti-CGRP monoclonal antibodies, Real-world, Treatment failure, Migraine

## Abstract

**Background:**

Atogepant is approved for migraine prevention and has shown strong efficacy in clinical trials. However, its effectiveness following failure of anti-CGRP monoclonal antibodies (MAbs) has not been evaluated in large real-world populations.

**Methods:**

This multicenter observational study conducted across Spanish headache units included adults with migraine who initiated atogepant after failure of ≥ 1 anti-CGRP MAb and had ≥ 3 months of follow-up. Baseline demographic and clinical variables were collected prospectively, with follow-up assessments at months 3 and 6. The primary outcome was the proportion of patients achieving a ≥ 50% reduction in monthly migraine days (MMD) at three months. Secondary outcomes included ≥ 30%, ≥ 75%, and 100% response rates; changes in headache days, pain intensity, acute medication use, and patient-reported outcomes; adverse events; treatment persistence; and factors associated with response.

**Results:**

A total of 252 patients were included (mean age 48.9 ± 12 years; 83.3% female; 80.6% with chronic migraine; 45.6% with continuous daily headache). Prior to atogepant, 39.7% had failed one anti-CGRP MAb, 27.0% two, 20.2% three, and 13.1% four. Median baseline MMD was 16, monthly headache days 27, and acute medication days 20. At 3 months, 44.4% achieved a ≥ 30% reduction in MMD, 29.7% ≥50%, and 11.7% ≥75%. Adverse events were reported in 52.5% of patients, most commonly constipation (30%) and nausea (25%). At three months, 26.2% had discontinued treatment (65.1% due to inefficacy, 28.8% due to intolerance). Treatment persistence at 180 days was 61% (95% CI 54 to 69%). A higher number of previously failed MAbs was independently associated with reduced odds of ≥ 50% response (RR 0.79, 95% CI 0.64 to 0.97). Moreover, a higher number of previously failed MAbs was associated with diminished improvements across multiple clinical endpoints, including headache frequency, intensity, acute medication use, and disability measures.

**Conclusion:**

Atogepant may represent a viable treatment option for patients with migraine who have failed anti-CGRP MAbs. In this large real-world cohort, approximately one-third of patients achieved a ≥ 50% response, despite a treatment-refractory profile. However, the likelihood of response decreases with a higher number of previously failed MAbs, and mild adverse events are frequent.

**Supplementary Information:**

The online version contains supplementary material available at 10.1186/s10194-025-02239-1.

## Highlights

This is the largest real-world study to date evaluating atogepant after failure of anti-CGRP monoclonal antibodies in migraine, including a highly refractory multicenter cohort.

Atogepant after anti-CGRP monoclonal antibodies failure achieved a ≥ 50% response in 29.7% of patients and a ≥ 30% response in 44.4% at 3 months, with mild adverse effects reported in over half of cases.

A higher number of previously failed MAbs, trigeminoautonomic symptoms, and depression were independently associated with lower likelihood of response.

**Supplementary Information:**

The online version contains supplementary material available at 10.1186/s10194-025-02239-1.

## Introduction

The emergence of anti-CGRP monoclonal antibodies (MAbs) has transformed the preventive treatment paradigm for migraine due to their high efficacy and tolerability [1]. These benefits extend to patients who have failed prior preventive therapies [2]. Furthermore, real-world data indicates that approximately 60% of patients achieve a ≥ 50% reduction in monthly migraine days [3]. Nonetheless, a proportion of patients remain partial responders or non-responders, creating a therapeutic dilemma regarding whether to switch to another MAb or consider moving to newly commercialized gepants [4].

Atogepant, a gepant approved for the preventive treatment of migraine, has demonstrated robust efficacy and favorable tolerability in patients with both episodic [5–7] and chronic migraine [8]. It has also shown promising results in subpopulations with prior failure to oral preventive treatments [9]. However, no clinical trial has specifically assessed its use in patients previously treated with anti-CGRP MAbs, which poses a critical clinical question, as there is currently no established guidance on the therapeutic approach following MAb failure.

Two early Italian real-world studies evaluating atogepant also included patients who had previously failed anti-CGRP MAbs [10, 11]. In these studies, overall response rates were consistent with, or even higher than, those reported in clinical trials, with 56.5% and 65.9% of patients, respectively, achieving a ≥ 50% reduction in migraine days. Among patients with prior MAb failure, who represented 52.8% (*n* = 56) and 41.5% (*n* = 34) of the respective cohorts, ≥ 50% response rates were modestly lower (46.4% and 52.9%). However, these studies included a limited number of patients with prior MAb failure (90 across both studies) and did not specifically focus on this population.

Later, a single-center Spanish study reported on 44 patients who initiated atogepant following MAb failure, with ≥ 50% and ≥ 30% response rates of 18.2% and 25%, respectively, and adverse effects in 50% of patients [12]. While these findings suggest a potential benefit in a subset of patients, their generalizability is limited by the small sample size.

Therefore, this multicenter Spanish study aimed to evaluate the effectiveness, tolerability, treatment persistence, and factors associated with response to atogepant in a large cohort of patients with migraine who had previously failed anti-CGRP monoclonal antibodies.

## Methods

### Patients

This observational retrospective study analysed clinical data that were prospectively collected in routine practice across 17 headache units in Spain. Patients who initiated atogepant between June 2024 and January 2025 after having failed at least one anti-CGRP MAb were included. Eligibility criteria required fulfilment of the ICHD-3 (International Classification of Headache Disorders, 3rd edition) diagnostic criteria for migraine [13] and a minimum follow-up period of three months.

Inclusion of patients with at least one prior anti-CGRP MAb failure was motivated by the exclusion of this subgroup from clinical trials, while also allowing patients with multiple prior MAb failures to more accurately reflect real-world clinical practice.

In accordance with the Spanish government’s reimbursement criteria for atogepant, all enrolled patients had ≥ 8 monthly migraine days at baseline and had failed at least three preventive treatments, including onabotulinumtoxinA (BTX-A) in the case of chronic migraine. Atogepant was prescribed as a daily oral treatment. Treatment decisions were made individually by the attending physician, taking into account concomitant use of oral preventives or BTX-A. Patients with medication-overuse headache (MOH) were not required to undergo a specific withdrawal strategy prior to atogepant initiation. Comorbidities such as depression, anxiety, and fibromyalgia were recorded based on a previously established clinical diagnosis documented at the baseline visit; no formal diagnostic criteria were reassessed for this study.

### Variables

All clinical data were prospectively collected from the initiation of atogepant treatment, with quarterly scheduled visits and a minimum follow-up of three months. Baseline variables collected included age, sex, migraine duration, time since chronification (if applicable), phenotypic traits (aura, unilateral pain, unilateral trigeminoautonomic symptoms, nausea/vomiting, photophonophobia, and allodynia), prior preventive treatments including MAbs (with documentation of duration and reason for discontinuation), concomitant oral preventives or BTX-A, and comorbidities (depression, anxiety, fibromyalgia).

Quarterly assessments included monthly migraine days (MMD), monthly headache days (MHD), frequency of monthly headache days by maximum pain intensity (none, mild, moderate, or severe on a 4-point scale), acute medication days per month (AMDM), the 6-item Headache Impact Test (HIT-6), the Migraine Disability Assessment (MIDAS, with higher scores reflecting greater disability), Patients’ Global Impression of Improvement (PGII; 7-point scale where 1 = no change and 7 = very much better) or Patients’ Global Impression of Change (PGIC; 7-point scale where 1 = very much better and 7 = very much worse), the Hospital Anxiety and Depression Scale (HADS, with higher scores reflecting greater anxiety/depression), the Migraine-Specific Quality of Life (MSQ, with lower scores reflecting worse migraine-related quality of life), adverse effects, and time to treatment discontinuation along with reasons.

Headache parameters (MMD, MHD, AMDM, and headache intensity) were recorded using standardized paper or electronic headache diaries, in accordance with each center’s routine clinical practice. A headache day was defined as any calendar day with a headache episode. A migraine day was defined as a headache lasting ≥ 4 h that met ICHD-3 criteria for migraine or probable migraine, or a headache successfully treated with a triptan, ergotamine, or other migraine-specific acute medication. Patient-reported outcomes were collected using standardized paper or electronic forms at each visit. Although the participating centers did not follow a specific protocol for this study, clinical variables were prospectively collected in line with the consensus recommendations of the Headache Study Group of the Spanish Society of Neurology and were subsequently used for analysis.

### Outcome measures

The primary endpoint was the proportion of patients achieving a ≥ 50% reduction in MMD at 3 months. Secondary endpoints included the proportion of patients achieving a ≥ 50% response at 6 months, as well as the proportion of patients achieving ≥ 30%, ≥ 75%, and 100% responses at 3 and 6 months. Additional secondary outcomes included changes in MMD, MHD, AMDM, headache intensity, and patient-reported outcomes (HIT-6, MIDAS, HADS, MSQ) after 3 and 6 months and adjusted by prior MAbs. PGII and PGIC were also assessed at these time points. Adverse effects, retention rates, time to treatment discontinuation, and factors associated with ≥ 50% and ≥ 30% response were evaluated. Subgroup analyses were conducted to compare outcomes based on migraine type (episodic vs. chronic), number of previously failed MAbs, prior erenumab use, and the presence at baseline of continuous daily headache (CDH, defined as headache occurring every day of the month) or MOH. Results were also stratified by washout duration, defined as short (≤ 90 days) or long (> 90 days) according to the time between the last administered MAb and atogepant initiation.

For response-rate analyses (≥ 30%, ≥ 50%, ≥ 75%, and 100% reduction in MMD), patients who discontinued atogepant before the 6-month visit were considered non-responders at month 6. In contrast, analyses of tolerability and patient-reported outcomes (HIT-6, MIDAS, HADS, MSQ, PGI) at each time point were restricted to patients with available assessments (complete cases).

### Statistical analysis

Demographic and clinical characteristics of the included population were summarized. For the continuous variables, mean and standard deviation, or median and interquartile ranges according to the distribution was used. For discrete variables, the corresponding absolute and relative frequencies were reported. Response rates and adverse effects were calculated using absolute values and proportions.

To examine the evolution of relevant clinical endpoints independent linear quantile mixed models were estimated for each endpoint with time as a fixed factor and patient as a random factor. These models were repeated adjusted by previous MAbs. The interaction between time and previous MAbs was also tested.

For the analysis of time to treatment discontinuation, a Kaplan-Meier curve was plotted.

To study the factors associated with treatment response > = 30% and > = 50% in MMD, independent logbinomial models were performed for each outcome and factor. These models were repeated adjusted for known factors. Multiple imputation by chained equations with fifty iterations of imputation was applied to account for missing data in MIDAS, AMDM, HIT6 and TMC measures at baseline [14, 15]. The assumption that unobserved values were missing at random was deemed to be appropriate because we could not find any pattern among the missing values [16].

Statistical tests were performed at a 5% significance level and were bilateral. The statistical packages R 4.4.1 or higher for Windows version and SPSS version 20 (IBM) were used for data analysis and processing. No formal sample size calculation was conducted, as the number of patients was determined by available data across participating headache units, in line with the exploratory and hypothesis-generating nature of this real-world study. No adjustments were applied to account for potential inter-center variability.

### Ethics approval and consent to participate

The study was approved by the Ethical Committee of the coordinating centre with reference EOM018/25, which granted a waiver of informed consent due to the retrospective nature of the study. The confidential information of the patients was handled in accordance with Spanish regulations.

## Results

### Baseline characteristics

**Fig. 1:**
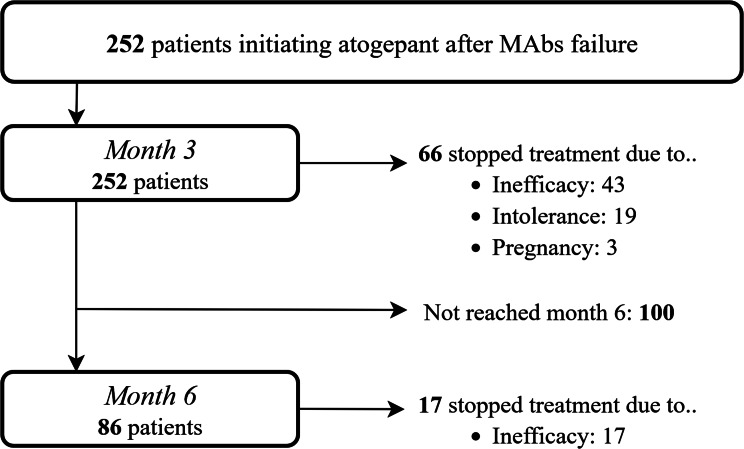
Flowchart for patient inclusion. MAbs = monoclonal antibodies

Of the 252 included patients who initiated atogepant and had more than three months of follow-up, 86 had also available data at month 6 (see Flowchart in Fig. 1). Patients who discontinued treatment before month 6 were considered non-responders in the effectiveness analysis at month 6, resulting in a total of 152 patients included in this response rate analysis. In contrast, tolerability outcomes and functional scales at month 6 were evaluated exclusively in the 86 patients with available data.

The mean age was 48.9 (SD 12.7) years, with 210 (83.3%) patients being women. The median duration of migraine was 29 years (interquartile range [IQR] 17 to 40), and the median duration since chronification was 10 years (IQR 5 to 15). Phenotypic characteristics, comorbidities, and prior preventive treatments are summarized in Table 1. Atogepant 60 mg was prescribed to all patients except for two (0.8%) who received 10 mg.

**Table 1:** Demographic and phenotypic characteristics, comorbidities, and prior preventive treatments

*N*	252
**Women [n (%)]**	210 (83.3)
**Age [mean ± SD]**	48.9 ± 12.7
**Phenotypic characteristics [n (%)]**	
Aura	76 (30.2)
Unilateral pain	141/236 (59.7)
Unilateral trigeminoautonomic symptoms	48/236 (20.3)
Nausea or vomiting	188/236 (79.7)
Photophonophobia	208/236 (88.1)
Allodynia	114/219 (52.1)
**Comorbidities [n (%)]**	
Anxiety	118/240 (49.2)
Depression	97/240 (40.4)
Fibromyalgia	34/240 (14.2)
**Prior preventive treatment classes [median (IQR)]**	5 (5–6)
**Prior oral preventive treatments [n (%)]**	
Beta-blockers	176 (69.8)
Topiramate	208 (82.5)
Amitriptyline	212/235 (90.2)
Flunarizine	158 (62.7)
Lisinopril or candesartan	119/251 (47.4)
**Prior treatment with BTX-A [n (%)]**	228 (90.5)
**Prior treatment with anti-CGRP MAbs [n (%)]**	
Erenumab	130 (51.6)
Galcanezumab	165 (65.5)
Fremanezumab	159 (63.1)
Eptinezumab	67 (26.6)

IQR = interquartile range; SD = standard deviation

Variables with missing data are indicated within the table

Patients had failed a median of 2 (IQR 1–3) MAbs. Specifically, 100 patients (39.7%) had failed one MAb, 68 (27.0%) two MAbs, 51 (20.2%) three MAbs, and 33 (13.1%) four MAbs. The median time since initiation of the first MAb was 2.58 years (IQR 1.29–3.87), with a baseline MMD of 16.5 (IQR 12–24) at that time. The last MAb was discontinued a median of 94 days prior (IQR 28.5–426), primarily due to inefficacy (86.9%), followed by intolerance (8.4%).

At baseline, 80.6% of patients had CM and 45.6% had CDH. The median MMD was 16 (IQR 11–25), MHD was 27 (IQR 18.25–30), and AMDM was 20 (IQR 13–30). Median patient-reported outcomes were as follows: HIT-6 70 (IQR 65–74); MIDAS 79.5 (IQR 45–120); HAD-A 9 (IQR 6–13); HAD-D 8 (IQR 4–13); and MSQ 21.2 (IQR 15–45.4). Clinical variables at baseline, month 3, and month 6 are detailed in Table S1 (Supplementary Material).

### Effectiveness

**Fig. 2:**
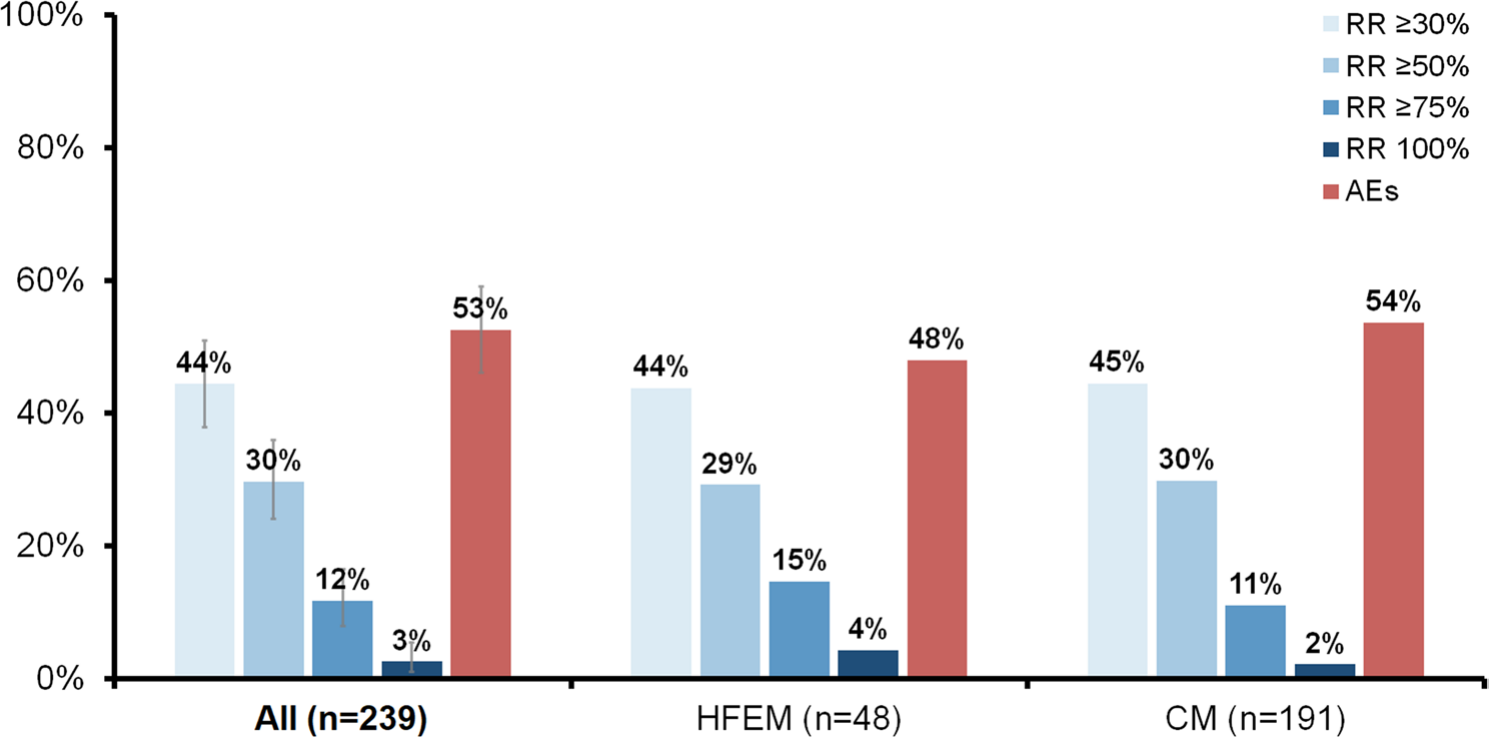
Response rates and adverse effects at three months for the overall cohort and for HFEM and CM subgroups. AEs = adverse effects; CM = chronic migraine; HFEM = high-frequency episodic migraine; RR = response rate. Confidence intervals 95%

At three months, 29.7% of patients achieved a ≥ 50% reduction in MMD. The proportion of responders for each percentage threshold is shown in Fig. 2, along with responder rates for high-frequency episodic migraine (HFEM) and chronic migraine (CM). At six months, the overall proportions of responders were 28.9% for ≥ 30% reduction, 21.1% for ≥ 50% reduction, 9.2% for ≥ 75% reduction, and 2.6% for 100% reduction in MMD (*n* = 152, with patients who had discontinued counted as non-responders). A sensitivity analysis including only patients who reached month 6 (*n* = 86) showed 51.2% responders for ≥ 30% reduction, 37.2% for ≥ 50% reduction, 16.3% for ≥ 75% reduction, and 4.7% for 100% reduction.

The baseline median MMD of 16 days (IQR 11–25) was reduced to 11 (6–20) at 3 months and 10 (5–20) at 6 months (*n* = 86, patients with available data at month 6). Similarly, median MHD of 27 (18–30) was reduced to 19 (10–30) at 3 months and 18 (10–30) at 6 months. Changes in AMDM, headache days by intensity, HIT-6, MIDAS, HADS, MSQ scores, and PGIC and PGII values are shown in Table 1S (Supplementary Material). Patient-reported global impression of change at 3 months is graphically presented in Fig. 1S (Supplementary Material). Overall, 33% of patients rated themselves as “Much improved” or better on the PGI-C (*n* = 169), and 33% rated “Somewhat better” or more on the PGI-I (*n* = 97).

In mixed-effects quantile regression models, clinical outcomes at 3 and 6 months were evaluated relative to baseline using three models: (1) an unadjusted model assessing the effect of time; (2) a model adjusted for the number of prior anti-CGRP MAb failures (1 vs. ≥2); and (3) a model incorporating an interaction term between time and the number of prior MAbs.

For MMD, model 1 showed a median reduction of − 3.81 days at 3 months (95% CI − 5.07 to − 2.54; *p* < 0.001) and − 5.08 days at 6 months (95% CI − 7.09 to − 3.07; *p* < 0.001). Model 2 confirmed a significant main effect of having ≥ 2 prior MAbs (+ 6.44 days; 95% CI 4.11 to 8.78; *p* < 0.001). In model 3, patients with only one prior MAb had a median MMD reduction of − 7.44 days at 6 months (95% CI − 9.72 to − 5.16; *p* < 0.001), while the interaction term for ≥ 2 versus 1 prior Mab indicated a significantly smaller benefit, resulting in a net reduction of − 2.99 days in the ≥ 2 MABs group. Results for all other variables are presented in Table 2S (Supplementary Material).

**Fig. 3:**
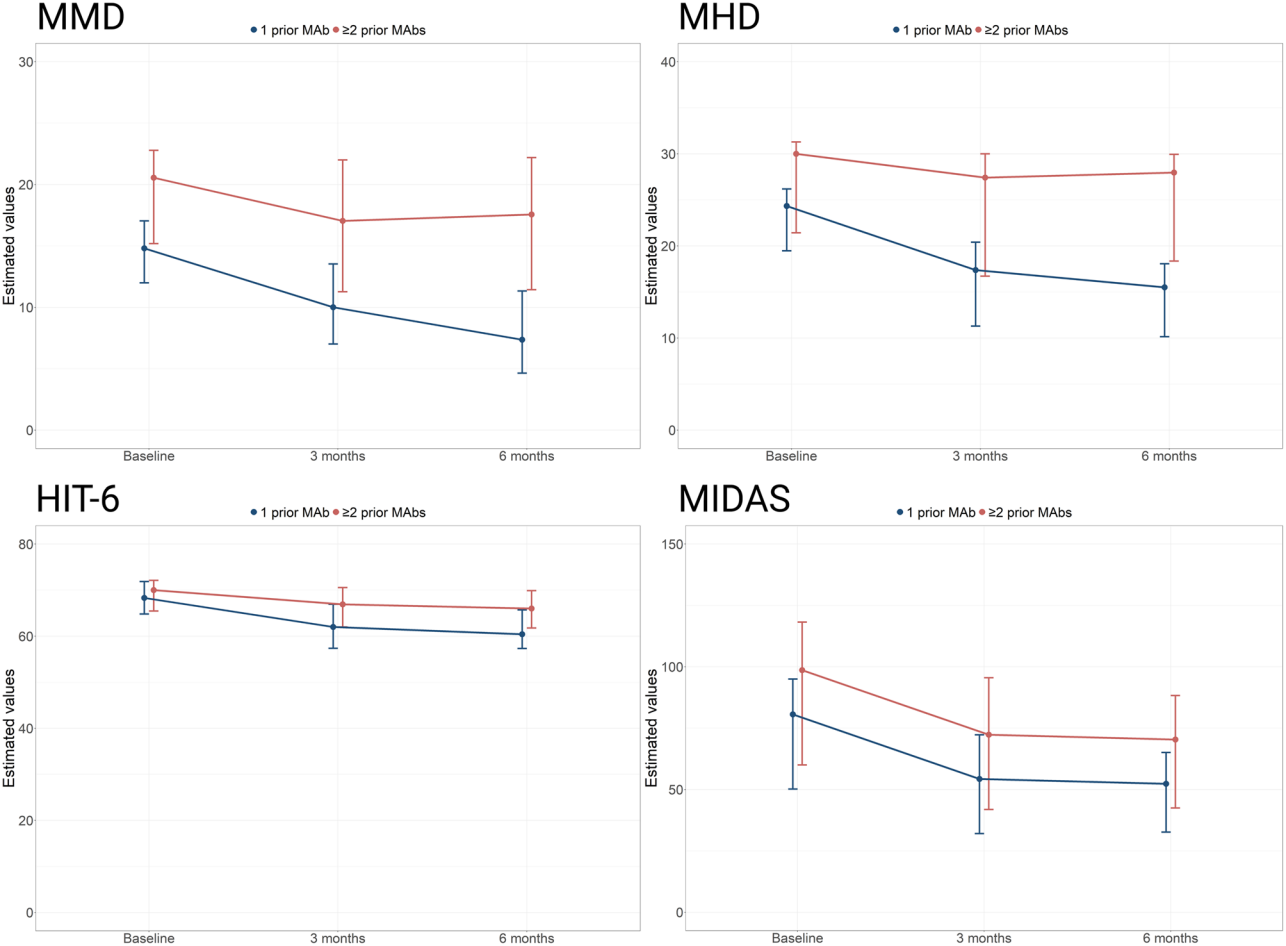
Median changes during follow-up in MMD, MHD and scores on the HIT-6 and MIDAS scales adjusted by previous MAbs (marginal effects model). MAbs = monoclonal antibodies; MHD = monthly headache days; MMD = monthly migraine days

Over the 6-month follow-up, atogepant was associated with statistically significant improvements in MMD, MHD, AMDM, headache intensity levels, HIT-6, and MIDAS scores. However, patients with ≥ 2 prior MAbs failures experienced significantly smaller improvements. In contrast, HADS-A, HADS-D and MSQ did not significantly differ based on prior MAbs history. Median changes across follow-up derived from the marginal effects models are presented in Figs. 2S and 3 (Supplementary Material).

### Tolerability

Adverse effects were reported in 52.5% of patients at month 3 and in 37.0% of patients at month 6 (also shown in Fig. 2). The distribution of reported adverse effects is detailed in Table 2. By month 3, 7.9% of patients had discontinued treatment due to intolerance. Among these, the most common reasons were nausea (63.2%), constipation (36.8%), epigastric pain (10.5%), and dizziness (5.3%), with six patients reporting more than one adverse effect. No treatment discontinuations due to adverse events occurred at month 6.

**Table 2:** Adverse effects reported after 3 and 6 months of Atogepant treatment

Adverse effects [*n* (%)]	M3 (*n* = 240)	M6 (*n* = 81)
Any	126 (52.5)	30 (37)
Constipation	72 (30)	14 (17.3)
Nausea	60 (25)	12 (14.8)
Fatigue	14 (5.8)	3 (3.7)
Weight or appetite loss	12 (5)	6 (7.4)
Somnolence	8 (3.3)	4 (4.9)
Epigastric pain/dyspepsia	8 (3.3)	0
Dizziness	7 (2.9)	1 (1.2)

### Persistence

Treatment discontinuation occurred in 66 patients (26.2%) by month 3, mainly due to inefficacy (65.1%), followed by intolerance (28.8%), pregnancy (4.5%), and other reasons (1.5%). At month 6, 17 patients (19.8%) discontinued treatment, all due to inefficacy.

**Fig. 4:**
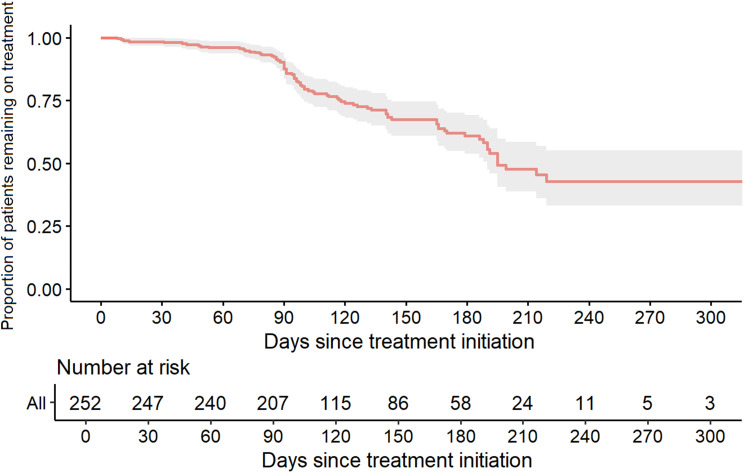
Kaplan-Meier survival analysis showing treatment retention rates during follow-up

Overall, during follow-up, 32.5% of patients discontinued treatment, with a median time to discontinuation of 97.5 days (IQR 86–141). The survival analysis is shown in Fig. 4. The probability of continuing treatment at 90 days was 88% (95% CI 83–92%), and at 180 days was 61% (95% CI 54–69%).

### Factors associated with response

Regarding baseline variables associated with a ≥ 50% response at three months, prior anti-CGRP MAb treatment was significantly associated with a lower likelihood of response (RR 0.79; 95% CI 0.65–0.97). The presence of unilateral trigeminoautonomic symptoms was also associated with reduced response (RR 0.51; 95% CI 0.26–1.00; *p* = 0.047). In the multivariate model, only prior MAb treatment remained significantly associated with reduced response (RR 0.79; 95% CI 0.64–0.97).

For a ≥ 30% response at three months, prior MAb treatment (RR 0.83; 95% CI 0.72–0.96), previous use of erenumab (RR 0.69; 95% CI 0.52–0.92), trigeminoautonomic symptoms (RR 0.61; 95% CI 0.39–0.97), depression (RR 0.66; 95% CI 0.48–0.91), and anxiety (RR 0.72; 95% CI 0.54–0.97) were all associated with a lower likelihood of response in bivariate analyses. In the multivariate analysis, factors associated with a lower likelihood of achieving a ≥ 30% response included prior MAb treatment (RR 0.83; 95% CI 0.73–0.95), trigeminoautonomic symptoms (RR 0.63; 95% CI 0.40–0.98), depression (RR 0.66; 95% CI 0.49–0.89), and higher baseline MMD (RR 0.98; 95% CI 0.97–1.00; *p* = 0.025). Conversely, longer duration since migraine chronification (RR 1.01; 95% CI 1.00–1.02; *p* = 0.018) and higher AMDM (RR 1.02; 95% CI 1.01–1.04) were associated with a greater likelihood of response.

**Fig. 5:**
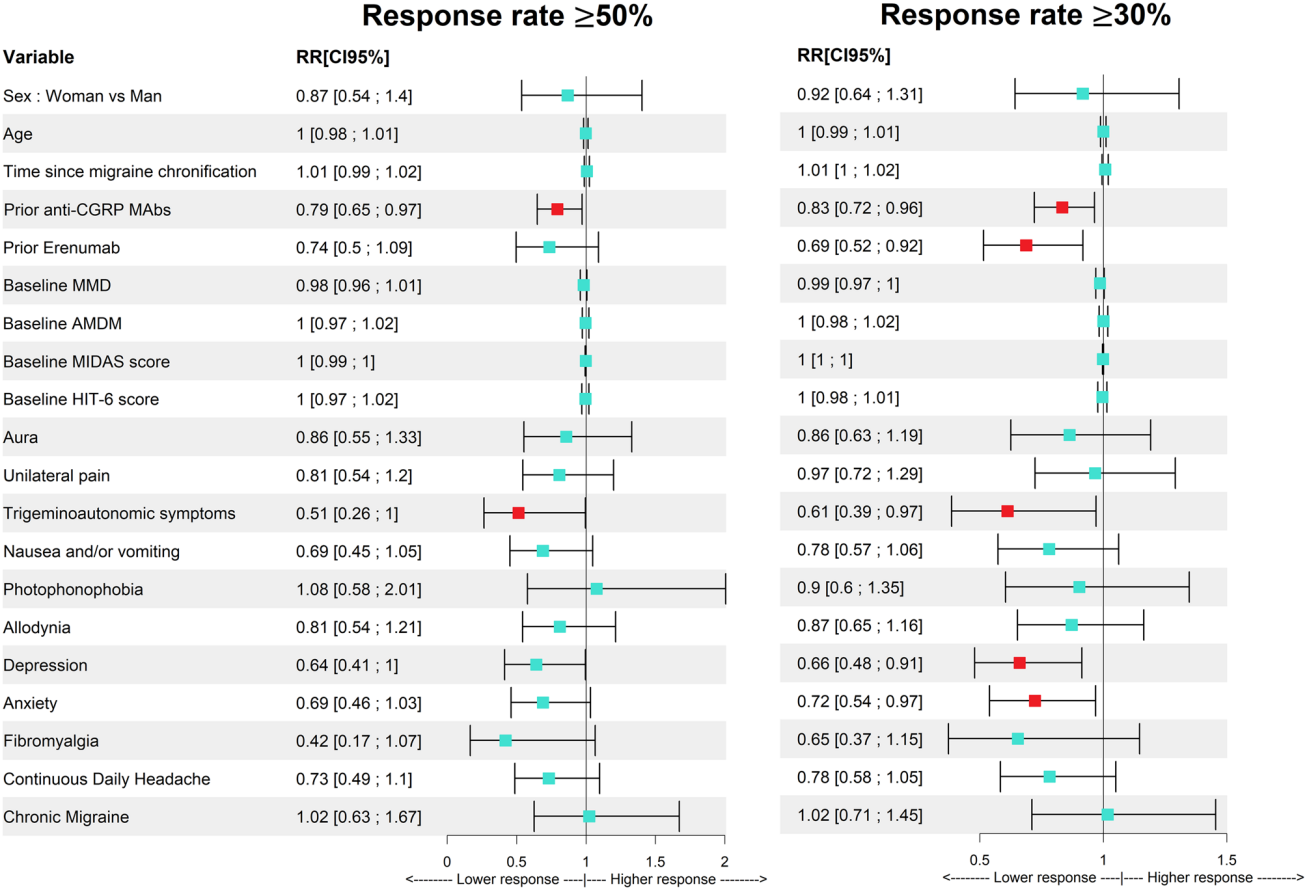
Relative risks for associations with a ≥ 50% and ≥ 30% response rates at three months (bivariate analysis). *Statistically significant variables are painted in red. MAbs = monoclonal antibodies; MMD = monthly migraine days; AMDM = acute medication days per month; MIDAS = Migraine Disability Assessment Scale; HIT-6 = Headache Impact Test; RR = relative risk. Confidence intervals represent 95% CI

Relative risks for the association with a ≥ 50% and ≥ 30% response at three months are shown in Fig. 5.

**Fig. 6:**
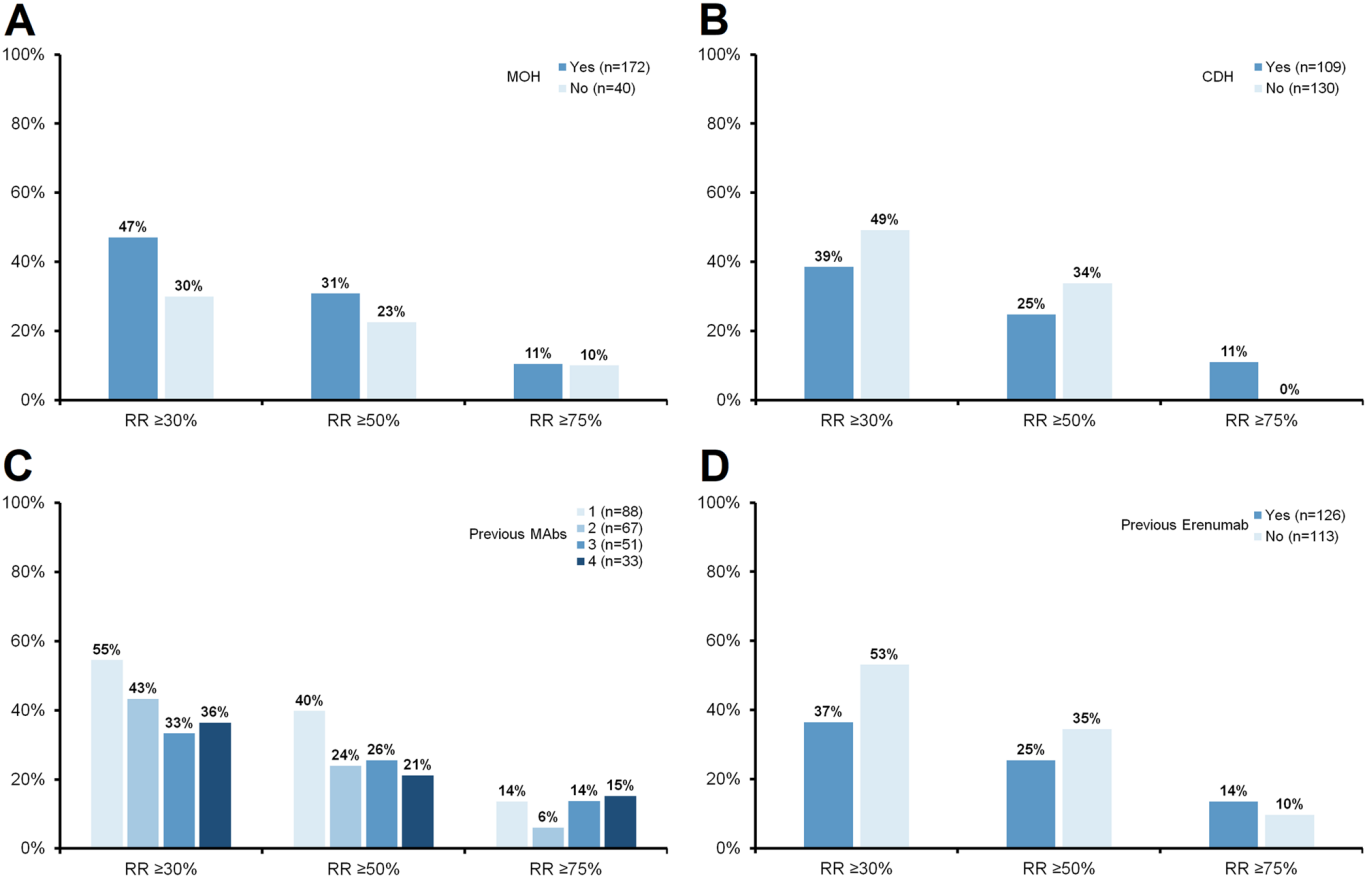
Response rates at three months by (A) baseline MOH, (B) baseline CDH, (C) number of prior MAbs, and (D) previous erenumab treatment. CDH = continuous daily headache; MAbs = monoclonal antibodies; MOH = medication overuse headache

Treatment response rates according to baseline MOH, baseline CDH, previous MAb treatment and previous erenumab use are presented in Fig. 6. Notably, at month 3, a ≥ 30% response rate was observed in 47% of patients with MOH versus 30% without, and in 39% of those with CDH versus 49% without. Among patients previously treated with erenumab, the ≥ 30% response rate was 37%, compared to 53% in erenumab-naïve individuals. Response rates stratified by the number of failed MAbs were 55%, 43%, 33%, and 36% for patients who had failed 1, 2, 3, and 4 MAbs, respectively.

Patients with a longer washout period (> 90 days since the last MAb, *n* = 118) showed response rates of 40.7%, 28.0%, 9.3%, and 2.5% for ≥ 30%, ≥ 50%, ≥ 75%, and 100% reduction in MMD, respectively. Those with a shorter washout period (≤ 90 days, *n* = 118) demonstrated similar rates of 47.5%, 31.4%, 13.6%, and 2.5%. Adverse effects were reported in 56.3% of patients in the long-washout group and 48.3% in the short-washout group.

## Discussion

In this real-world cohort of migraine patients with refractoriness to previous anti-CGRP MAb therapy, atogepant demonstrated a notable ≥ 50% response rate of 29.7%. Although this rate is logically lower than those observed in clinical trials involving patients who had failed only unspecific oral preventives [8, 9] these trials excluded patients with previous anti-CGRP therapies. Our findings reinforce the notion that targeting the CGRP pathway with an alternative agent can still yield clinical benefit in certain patients.

Reported adverse effects were mostly constipation (30%) and nausea (25%), occurring at higher rates than those reported in clinical trials of atogepant [17] but consistent with the initial real-world experience [10]. These adverse effects were mild and led to treatment discontinuation in only 7.9% of patients at three months. In contrast, the second real-world study [11] included only 41.5% of patients with MAb failure (with a minimum of more than five months since the last MAb), and while tolerability data were analyzed in the entire cohort (*n* = 183), only less than 50% (*n* = 82) of patients were actually assessed at month 3. Consequently, adverse effects may have been underestimated in that study, as they could have emerged later in patients not yet evaluated.

Compared with previous real-world cohorts of atogepant use [10, 11], our series likely represents a more treatment-refractory population, characterized by a higher median number of previously failed treatments (five classes, including anti-CGRP MAbs), and higher rates of chronic migraine (80.6%), medication overuse headache (80.7%), anxiety (49.2%), and depression (40.5%). This likely explains the lower observed response rates (≥ 50% response of 29.7%) compared with the subanalysis of anti-CGRP MAb failure in the STAR (46.4%) and GIANT (52.9%) studies. Additionally, in those studies, most patients had failed only one previous MAb (at least 19 patients had failed two in STAR, while only 11 patients had failed two MAbs in GIANT, with none failing three or more), whereas in our cohort, 60.3% of patients had failed two or more MAbs.

A recently published single-center cohort of 44 patients who initiated atogepant following MAb failure offers results more comparable to ours. In that study, the ≥ 50% and ≥ 30% response rates were 18.2% and 25%, respectively, while adverse effects were reported in 50% of patients [12]. The clinical characteristics of that cohort were similar to ours, with high rates of chronic migraine and medication overuse headache. However, important differences exist: erenumab exposure was notably lower (11.4% vs. 51.6% in our cohort), and fewer patients had failed more than one MAb (47.8% vs. 60.3%). Additionally, their primary outcome was based on monthly headache days (MHD) rather than monthly migraine days (MMD), which may have contributed to the lower reported response rates. When considering their data on monthly moderate-to-severe headache days (MSHD), the response rates are more aligned with ours: 47.6% achieved a ≥ 30% reduction and 33.3% achieved a ≥ 50% reduction.

In this highly refractory cohort—80.6% with chronic migraine and 45.6% with continuous daily headache—a ≥ 30% reduction in monthly migraine days, achieved by 44% of patients, may still represent a clinically meaningful improvement. Importantly, relying exclusively on frequency-based responder thresholds may underestimate treatment benefit, as disability, impact, and quality-of-life measures capture additional therapeutic effects [18]. Patient-reported outcomes are validated indicators of clinical response [19], and in the context of highly refractory migraine, where reductions in attack frequency are often modest, these parameters may be even more informative than frequency alone. Ultimately, these domains correlate well with changes in headache frequency [20] and support a multidimensional interpretation of atogepant effectiveness in this population.

The present study also aimed to identify potential predictors of response to atogepant in patients who had previously failed anti-CGRP therapy. As expected, a greater number of prior MAbs was consistently associated with a reduced likelihood of response—an observation that aligns with the concept that successive switches within the same anti-CGRP pathway tend to yield diminishing response rates [21]. Beyond response rates, our data also revealed smaller improvements in headache intensity, HIT-6 and MIDAS scores, and acute medication use among patients with ≥ 2 prior MAb failures compared to those who had failed only one. Taken together, these findings support the early use of atogepant after the first anti-CGRP MAb failure, as this strategy may increase the probability of achieving a favorable clinical response.

To date, switching between MAb types—whether targeting the CGRP ligand or receptor—has not been consistently associated with differential response rates [22–24]. However, in our study, prior erenumab use was associated with a lower likelihood of achieving a ≥ 30% response, with only 37% of patients previously treated with erenumab responding, compared to 53% among erenumab-naïve patients. This observation reopens the hypothesis that transitioning from a CGRP ligand-targeting MAb to a receptor antagonist—such as atogepant—may confer added benefit in some patients.

Interestingly, the presence of unilateral trigeminoautonomic symptoms was also associated with a lower response. This finding contrasts with previous literature suggesting such symptoms may indicate a more responsive phenotype due to trigeminovascular activation [25, 26]. However, in this refractory population, these symptoms may reflect alternative or overlapping pathophysiological mechanisms, such as PACAP-mediated pathways [27, 28], which are not targeted by CGRP antagonism.

Depression was likewise associated with a lower probability of response, consistent with prior evidence showing reduced treatment efficacy in patients with psychiatric comorbidities [29].

Lastly, MMD, AMDM, and time since chronification were also associated with a ≥ 30% response in the multivariate analysis. However, given the small effect sizes and borderline statistical significance, these associations should be interpreted with caution, as they may reflect chance findings or artifacts related to model specification rather than true predictive factors.

The present cohort included specific subpopulations such as patients with CM, CDH and MOH. None of these factors were associated with differential response rates in our analysis. As illustrated in Figs. 2 and 6, the response in these subgroups does not appear to be significantly lower, suggesting that such patients should not be excluded from a therapeutic trial of atogepant, even in the context of refractoriness to MAbs. Likewise, washout duration between the last MAb and atogepant initiation did not influence the observed treatment effect.

This study represents a significant contribution, providing robust real-world evidence on the effectiveness of atogepant following anti-CGRP MAb failure in a large, multicenter cohort. It addresses a critical clinical question regarding the utility of switching to a gepant after MAb refractoriness. However, several limitations should be acknowledged. The retrospective and multicenter nature of the study inherently carries a risk of missing data, inaccurate variable reporting, and potential information bias related to inter-center variability. Moreover, the absence of a control group precludes direct comparisons to alternative therapeutic strategies. Given that the included population represents a highly treatment-refractory subset—with high rates of CM, CDH, and medication overuse—the observed response rates may underestimate the efficacy of atogepant in less severe or earlier-stage cases. Additionally, the relatively short follow-up period may limit the ability to capture long-term treatment effects and sustained benefits. Future studies with extended follow-up durations (e.g., 12 months) are essential to evaluate long-term persistence, delayed onset of efficacy, and potential tolerability issues.

## Conclusion

Atogepant could be a valid therapeutic option for patients with migraine who have failed prior anti-CGRP MAb treatments, demonstrating a ≥ 50% response rate in approximately one-third of cases. Mild adverse effects were reported in up to 50% of patients, most commonly constipation and nausea. Notably, the number of previously failed MAbs was inversely associated with the likelihood of response, highlighting that greater prior exposure to anti-CGRP MAbs is associated with lower effectiveness of atogepant.

## Supplementary Information

Below is the link to the electronic supplementary material.

Supplementary Material 1

Supplementary Material 2

Supplementary Material 3

## Data Availability

The datasets used and/or analysed during this study are available from the corresponding author on reasonable request from any qualified investigator.
