# Supplementary figures and images for: Atogepant after anti-CGRP monoclonal antibodies failure in migraine: a multicenter real-world study of effectiveness, safety, persistence and predictors of response
Source: J Headache Pain. 2025 Nov 28;27(1):2. doi: 10.1186/s10194-025-02239-1 (PMC12764079; doi:10.1186/s10194-025-02239-1)

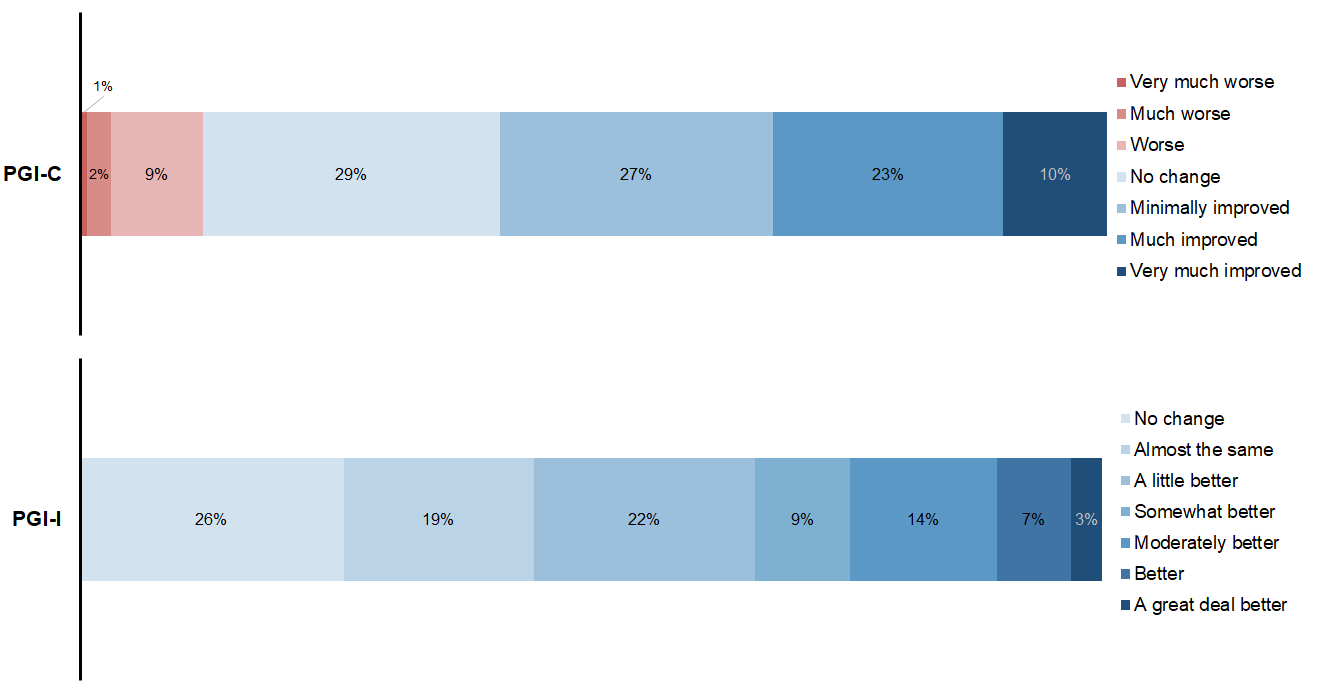

Supplement: Supplementary file 1 — Supplementary Material 1 [file 10194_2025_2239_MOESM1_ESM.png]

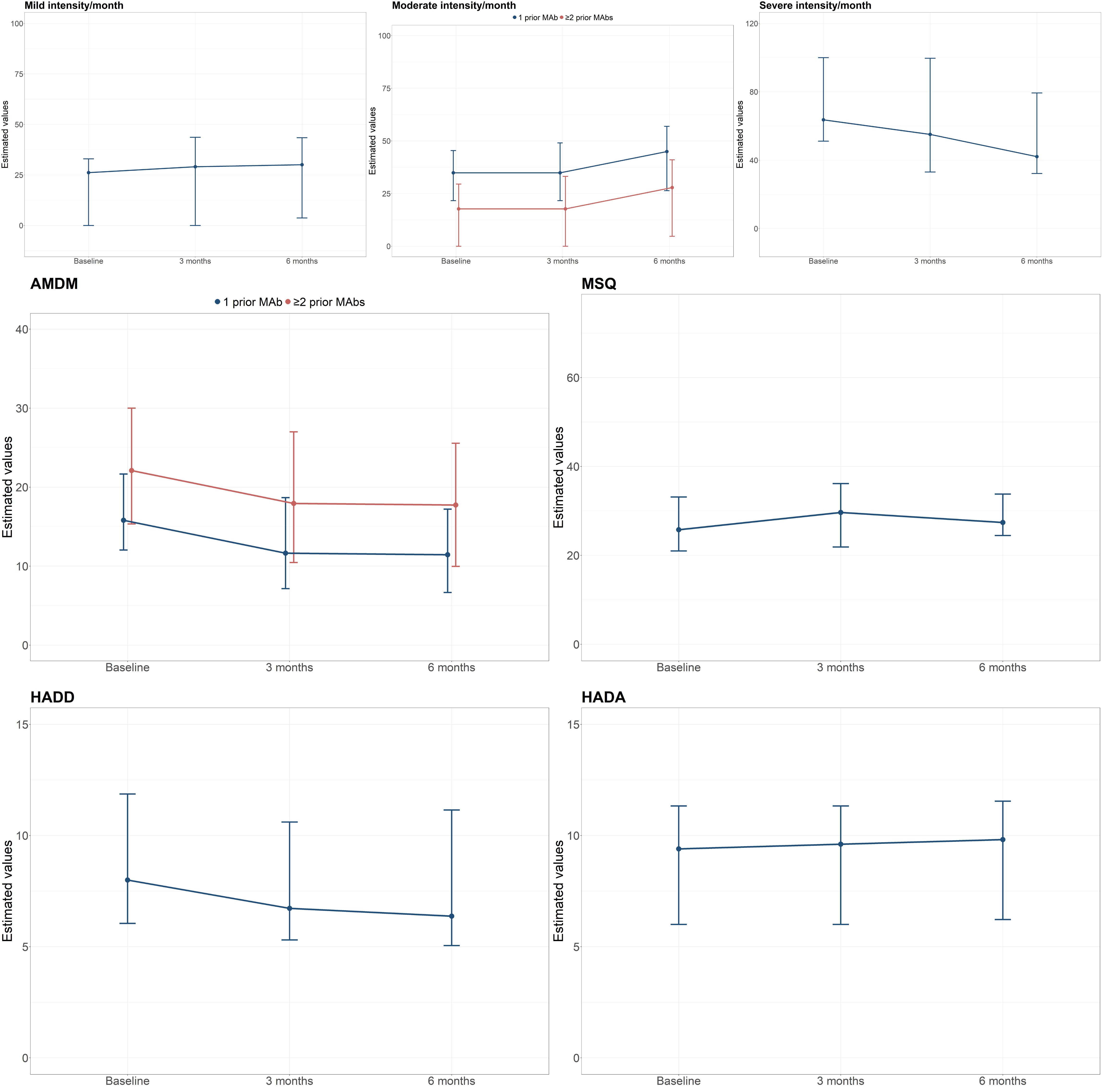

Supplement: Supplementary file 2 — Supplementary Material 2 [file 10194_2025_2239_MOESM2_ESM.png]
