# Supplementary material for: Atogepant after anti-CGRP monoclonal antibodies failure in migraine: a multicenter real-world study of effectiveness, safety, persistence and predictors of response
Source: J Headache Pain. 2025 Nov 28;27(1):2. doi: 10.1186/s10194-025-02239-1 (PMC12764079; doi:10.1186/s10194-025-02239-1)
Supplement: Supplementary file 3 — Supplementary Material 3 [file 10194_2025_2239_MOESM3_ESM.docx]

**Table 1S. Headache frequency, intensity, acute medication use, patient-reported outcomes (PROs), and concomitant treatment variables at baseline, month 3 (M3), and month 6 (M6).**

|  | Baseline | M3 | M6 |
| --- | --- | --- | --- |
| N | 252 | 239 | 86 |
| MMD [median (IQR)] | 16 (11-25) | 11 (6-20) | 10 (5-20) |
| MHD [median (IQR)] | 27 (18.3-30) | 19 (10-30) | 18 (10-30) |
| CM [n (%)] | 203 (80.6) | 130 (54.4) | 39 (45.3) |
| CDH [n (%)] | 115 (45.6) | 78 (32.6) | 26 (30.2) |
| Intensities [median (IQR)]  Mild days/month  Moderate days/month  Severe days/month | 1 (0-8) n=129/252  4 (0-10) n=113/252  11 (8-19.8) n=140/252 | 1 (0-8.25) n=130/239  3 (0-10) n=115/239  7 (2.5-13.5) n=141/239 | 0.5 (0-10.5) n=42/86  4 (0-10) n=35/86  4 (0-18.8) n=36/86 |
| AMDM [median (IQR)]  Triptans/ditans  NSAIDs | 20 (13-30) n=217/252  10 (4-15) n=210/252  14 (7-21) n=199/252 | 13 (7-25) n=201/239  8 (2-13) n=196/239  9 (3-18) n=185/239 | 12 (6-22) n=67/86  6 (0-12) n=68/86  7.5 (2-18) n=66/86 |
| Medication overuse [n (%)] | 180 (80.7) n=223/252 | 112 (53.3) n=210/239 | 35 (50) n=70/86 |
| HIT-6 [median (IQR)] | 70 (65-74) n=215/252 | 65 (60-72) n=186/239 | 63 (57-70) n=61/86 |
| MIDAS [median (IQR)] | 79.5 (45-120) n=142/252 | 42 (20-93) n=123/239 | 40 (12-70) n=43/86 |
| HADS [median (IQR)]  Anxiety  Depression | n=96/252  9 (6-13)  8 (4-13) | n=85/239  9 (5.5-13)  7 (3-12) | n=20/86  9.5 (7-13.8)  6 (3-13) |
| MSQ [median (IQR)] | 21.2 (15-45.4)  n=34/252 | 30 (18-56)  n=31/239 | 31.2 (9.3-59.9)  n=31/239 |
| PGII [median (IQR)]  PGIC [median (IQR)] | - | 3 (1-4.5)  n=97/239  3 (2-4)  n=169/239 | 3 (1.25-4)  n=32/86  3 (2-4)  n=54/86 |
| Concomitant oral treatment [n (%)] | 126 (53.8) n=234/252 | 123 (52.6) n=234/239 | 37 (48.7) n=76/86 |
| Concomitant BTX-A [n (%)] | 68 (27.8) n=245/252 | 68 (28.6) n=238/239 | 30 (35.3) n=85/86 |

AMDM = acute medication days per month; BTX-A = onabotulinum toxin A; CDH = continuous daily headache; CM = chronic migraine; HADS = Hospital Anxiety and Depression Scale; HIT6 = Headache Impact Test-6; IQR = interquartile range; MHD = monthly headache days; MIDAS = Migraine Disability Assessment; MMD = monthly migraine days; MOH = medication overuse headache; MSQ = Migraine-Specific Quality of Life Questionnaire; NSAIDs = non-steroidal anti-inflammatory drugs; PGIC = Patients’ Global Impression of Change.

Missing data are shown below each variable.

**Table 2S. Results of the mixed-effects quantile regression models for all assessed variables across the follow-up.**

Model 1: an unadjusted model assessing the effect of time.

Model 2: a model adjusted for the number of prior anti-CGRP MAb failures (1 vs. ≥2).

Model 3: a model incorporating an interaction term between time and the number of prior MAbs.

| MMD | |
| --- | --- |
| Model 1 | –3.81 days at 3 m (95% CI –5.07 to –2.54; p < 0.001) and –5.08 days at 6 m (–7.09 to –3.07; p < 0.001). |
| Model 2 | –4.14 days at 3 m (–5.19 to –3.10; p < 0.001) and –5.10 days at 6 m (–6.90 to –3.29; p < 0.001); main effect of ≥2 prior mAbs was +6.44 days (4.11–8.78; p < 0.001). |
| Model 3 | among those with one prior mAb, reduction at 6 m was –7.44 days (–9.72 to –5.16; p < 0.001); interaction term for ≥2 versus 1 prior mAb was +4.45 days (0.65–8.25; p = 0.026), yielding a net –2.99‐day reduction in the ≥2‐mAb group |
| MHD | |
| Model 1 | –3.93 days at 3 m (–6.14 to –1.72; p = 0.001) and –4.80 days at 6 m (–7.45 to –2.15; p = 0.001). |
| Model 2 | –4.71 days at 3 m (–6.98 to –2.43; p < 0.001) and –5.23 days at 6 m (–7.91 to –2.54; p < 0.001); main effect of ≥2 prior mAbs was +6.17 days (1.27–11.06; p = 0.017). |
| Model 3 | in the one-mAb group, –8.83 days at 6 m (–12.02 to –5.64; p < 0.001); interaction +6.79 days (3.21–10.36; p < 0.001), net –2.04 days for ≥2 mAbs. |
| AMDM | |
| Model 1 | –2.72 days at 3 m (–4.31 to –1.12; p = 0.002) and –4.49 days at 6 m (–7.13 to –1.86; p = 0.002). |
| Model 2 | –4.16 days at 3 m (–5.79 to –2.54; p < 0.001) and –4.36 days at 6 m (–6.37 to –2.34; p < 0.001); main effect of ≥2 mAbs +6.29 days (3.91–8.67; p < 0.001). |
| Model 3 | no significant interactions at either time point (p ≥ 0.158). |
| Headache intensities (in percentage of days) | |
| Mild intensity | no significant time or interaction effects in any model (all p > 0.05). |
| Moderate intensity | no significant time effects; Model 2 main effect of ≥2 mAbs –17.12% (–32.68 to –1.56; p = 0.035); no significant interactions. |
| Severe intensity |  |
| Model 1 | –8.54% at 3 m (p = 0.131), –21.54% at 6 m (–36.83 to –6.25; p = 0.008). |
| Model 2 | –13.03% at 3 m (–22.43 to –3.64; p = 0.009), –22.86% at 6 m (–38.19 to –7.54; p = 0.005). |
| Model 3 | in the one-mAb group –42.15% at 6 m (–59.92 to –24.38; p < 0.001); interaction +42.15% (19.68–64.63; p = 0.001), net 0% change in ≥2-mAb group. |
|  |  |
| HIT-6 | |
| Model 1 | –4.00 points at 3 m (–5.56 to –2.44; p < 0.001), –5.41 at 6 m (–7.29 to –3.53; p < 0.001). |
| Model 2 | –4.00 at 3 m (–5.44 to –2.56; p < 0.001), –5.60 at 6 m (–7.90 to –3.30; p < 0.001); no main effect of mAb number (p = 0.071). |
| Model 3 | –6.31 at 3 m (–8.65 to –3.96; p < 0.001), –7.89 at 6 m (–11.15 to –4.64; p < 0.001); interaction at 3 m +3.21 (0.12–6.31; p = 0.047), net –3.10 for ≥2 mAbs. |
| MIDAS | |
| Model 1 | –30.14 points at 3 m (–40.42 to –19.85; p < 0.001), –31.46 at 6 m (–39.82 to –23.11; p < 0.001). |
| Model 2 | –26.28 at 3 m (–37.07 to –15.48; p < 0.001), –28.25 at 6 m (–39.44 to –17.06; p < 0.001); main effect of ≥2 mAbs +18.03 (0.83–35.23; p = 0.045). |
| Model 3 | –27.68 at 3 m (–43.16 to –12.21; p = 0.001), –28.50 at 6 m (–44.42 to –12.58; p = 0.001); no significant interactions. |
| HADS: HADA (Anxiety) | |
|  | No significant time or interaction effects (all p > 0.05). |
| HADS: HADD (Depression) | |
| Model 1  Model 2 | –1.27 points at 3 m (–2.33 to –0.22; p = 0.021); 6 m change not significant.  –1.29 at 3 m (–2.26 to –0.32; p = 0.012); 6 m non-significant; no interactions. |
| MSQ | |
|  | No significant changes over time or interactions in any model (all p > 0.05). |

AMDM = acute medication days per month; HADS = Hospital Anxiety and Depression Scale; HIT6 = Headache Impact Test-6; MHD = monthly headache days; MIDAS = Migraine Disability Assessment; MMD = monthly migraine days; MSQ = Migraine-Specific Quality of Life Questionnaire.

**Figure 1S. Patients’ Global Impression of Improvement (PGI-I, n=97) and Patients’ Global Impression of Change (PGI-C, n=169) at three months, depending on availability at each center.**

**
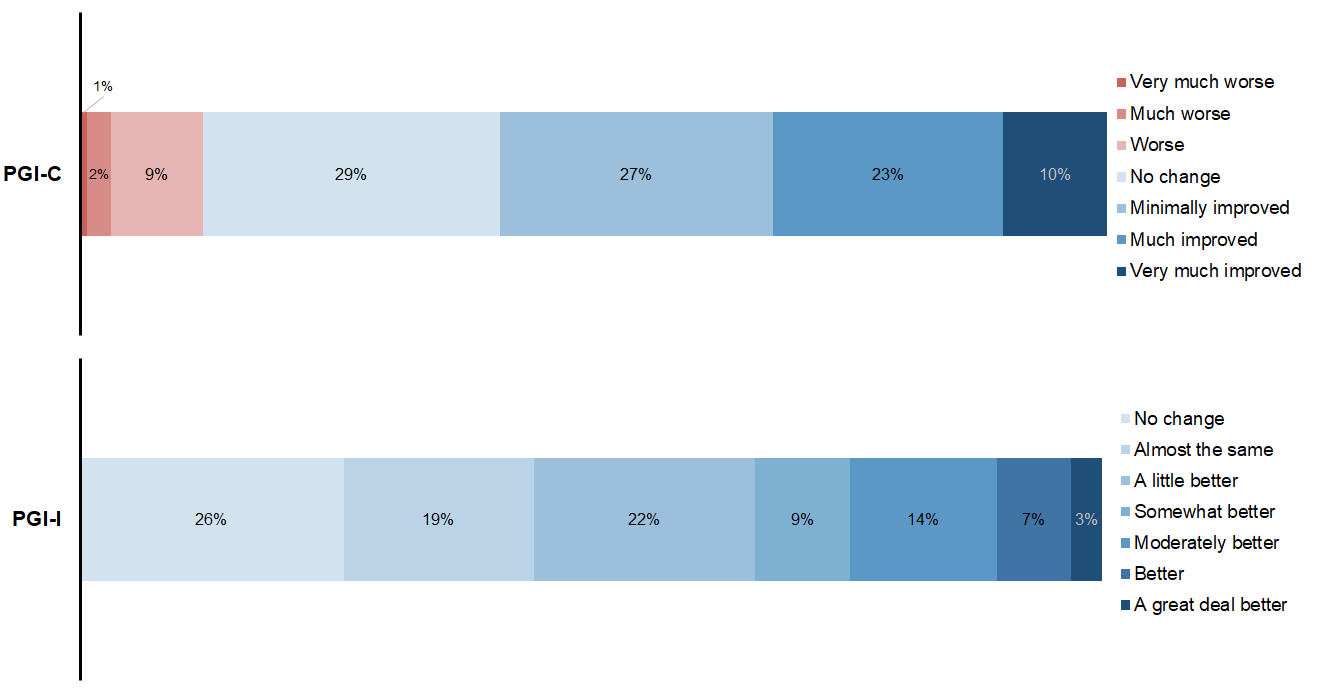
**

**Figure 2S. Median changes during follow-up in AMDM, headache intensities (percentage of days), HADS (HADA for anxiety and HADD for depression) and MSQ adjusted by previous MAbs (marginal effects model).**

**
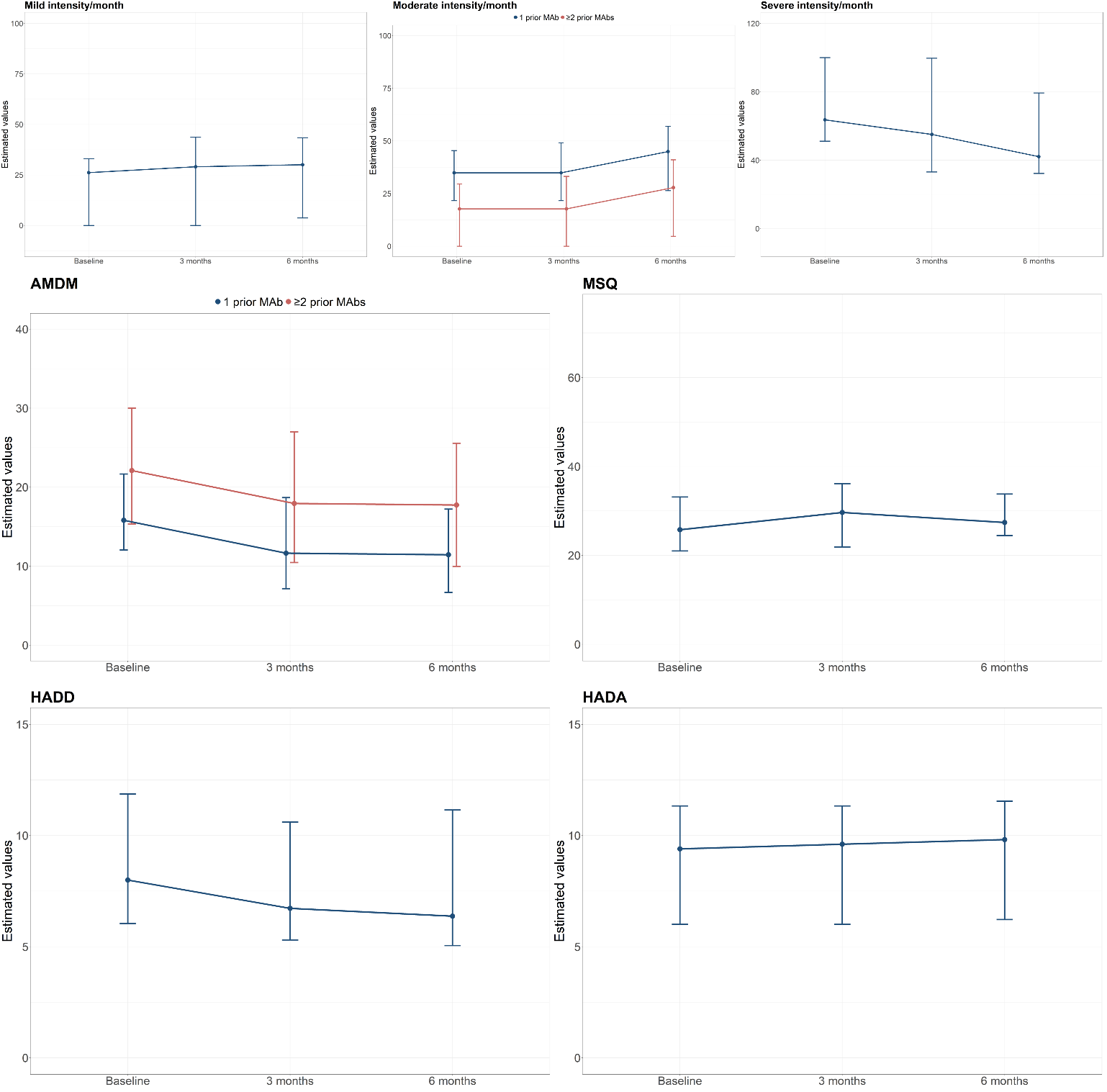
**
